# The Role of Attachment Anxiety and Avoidance in Predicting Proximal Minority Stressors among Gay and Lesbian People in Italy

**DOI:** 10.3390/ijerph21060655

**Published:** 2024-05-21

**Authors:** Tommaso Trombetta, Maria Noemi Paradiso, Fabrizio Santoniccolo, Luca Rollè

**Affiliations:** Department of Psychology, University of Turin, 10124 Turin, Italy; tommaso.trombetta@unito.it (T.T.); fabrizio.santoniccolo@unito.it (F.S.); l.rolle@unito.it (L.R.)

**Keywords:** adult attachment, minority stress, proximal minority stressors, internalized homonegativity, perceived discrimination

## Abstract

As has been widely documented, minority stress affects the psychosocial well-being of gay and lesbian people. Recently, researchers have turned their attention to psychological factors that may influence the level of minority stress experienced, in order to explain individual differences in perceptions of proximal minority stressors. The present research aimed at assessing the effect of attachment avoidance and anxiety on levels of perceived stigma and internalized homonegativity. A total of 163 participants who self-identified as lesbian or gay (*M_age_* = 32.56, *SD* = 10.87) were recruited and responded to the self-report questionnaires. Two multiple regression models were applied to assess the association between adult attachment and perceived stigma and internalized homonegativity. Results showed a positive association between attachment anxiety and avoidance and internalized homonegativity, as well as between attachment avoidance and perceived stigma. The emerging results demonstrate the impact of attachment anxiety and avoidance on proximal minority stressors and provide useful data for interventions addressing lesbian and gay people aimed at promoting security-based strategies of affect regulation and positive representations of self and others, which in turn may reduce the level of proximal minority stressors experienced and promote psychosocial well-being.

## 1. Introduction

Minority stress refers to the stressful condition that lesbian and gay people (LG) are exposed to because of their identification with a minority and socially stigmatized sexual orientation. It is a chronic stress related to the marginalization and discrimination that LG people experience in different social contexts, which negatively impact on psychosocial well-being [1,2,3,4,5]. The minority stress model was formulated by Meyer [2,5] to define the dimensions that contribute to minority stress and the lower mental health levels found in the LG population compared to the heterosexual population. It includes three major stress-inducing factors that move along a continuum from distal to proximal minority stressors. Distal minority stressors refer to the lived experience of discrimination, rejection and stigmatization based on one’s sexual orientation as a result of societal homonegative and heterosexist attitudes. Proximal stressors, on the other hand, include perceived stigma, i.e., the perception of being stigmatized because of one’s sexual orientation, and internalized homonegativity, i.e., negative attitudes and affects directed against oneself and one’s sexual identity as a consequence of internalizing socially conveyed homonegative attitudes.

Many studies over the decades have demonstrated the validity and usefulness of the minority stress model. Furthermore, numerous studies, reviews, and meta-analyses [6,7,8,9,10,11,12,13,14,15] have highlighted the negative impact of distal and proximal minority stressors on various indicators of individual and relational health, such as anxiety and depression symptoms, dysfunctional eating behaviors, suicidal ideation, couple well-being and adjustment, and Intimate Partner Violence (IPV). More recently, researchers turned their attention to understanding factors that may influence and modulate levels of minority stress experienced, with particular interest in predictors of internalized homonegativity and perceived stigma.

In this regard, attachment theory, and adult attachment in particular, may provide useful references for understanding individual differences in the perception of proximal minority stressors. Attachment theory has been developed by Bowlby [16,17,18] and focuses primarily on the attachment bond that is formed between a child and his or her caregiver from early infancy, promoting closeness in stressful conditions. Hazan and Shaver [19] first applied attachment theory to the bond between two adult partners. Building on the principles of attachment theory developed by Bowlby, they proposed the concept of adult attachment and defined it as the symmetrical, reciprocal bond between two partners that provides closeness and emotional regulation under stressful conditions. According to Mikulincer, Shaver, and collaborators [20,21,22,23], the attachment bond between two partners is influenced by the functioning of each partner’s adult attachment system, which can be measured by two dimensions: attachment anxiety and attachment avoidance. Individuals with higher levels of attachment anxiety tend to hyperactivate the attachment system. This results in reactivity to attachment needs and rejection signals from the partner, accompanied by vulnerability to negative affect, intense expression of one’s attachment needs, fear of abandonment, desire for closeness, and negative representations of oneself and of other people who are nonetheless perceived as desirable and potentially accessible. Individuals with higher levels of attachment avoidance, on the other hand, tend to deactivate the attachment system. This limits access of attachment needs in order to defend themselves against intolerant affect and the fear that their own demands for care will be met with rejection and inaccessibility from their partners. People with higher levels of attachment avoidance also show discomfort with closeness and fear of intimacy, accompanied by negative representations of the other and a seemingly positive, autonomous, and independent self-representation structured for defensive purposes to limit the emergence of attachment needs.

As proposed in the Integrated Attachment and Sexual Minority Stress Model (IASMSM) by Cook and Calebs [24], adult attachment and distal and proximal minority stressors can influence each other. Specifically, according to this model, adult attachment may influence an individual’s perceived level of minority stress, negatively impacting on well-being. Although there are few studies [25,26,27,28,29] that have examined the hypotheses formulated by Cook and Calebs’ [24] integrated model, the data identified have demonstrated an association between attachment anxiety and avoidance and the level of proximal minority stress experienced. More specifically, several studies [25,26,27,28,30] have shown a positive association between adult attachment and internalized homonegativity, while there are limited data on the impact of attachment anxiety and avoidance on perceived stigma. To the best of the authors’ knowledge, only one study [29] has investigated this association. It showed a positive association between attachment anxiety and perceived stigma and a negative association between attachment avoidance and perceived stigma. However, this latter association was no longer significant when the anxious dimension of adult attachment was not simultaneously included in the model. As suggested by the authors, this seems to indicate a partial overlap between the two dimensions of attachment and the role of possible moderating factors, underlining the need for further studies in this direction. Furthermore, the study by Zakalyk and Wei [29] only assessed perceived stigma, without considering other proximal minority stressors such as internalized homonegativity. Delving into the role of adult attachment in predicting different proximal minority stressors can be useful for theoretical and clinical purposes, in order to provide information for the development and implementation of interventions aimed at reducing levels of minority stress and promoting well-being in gay and lesbian people. These data are of particular value in Italy, where the civil rights of sexual minority people are still not fully recognized [31]. As shown by the 2024 Annual Review of the Human Rights Situation of Lesbian, Gay, Bisexual, Trans and Intersex People in Europe and Central Asia [32], Italy ranks 34th out of 49 European countries in terms of rights and inclusion. A recent report by the National Institute for Statistics (ISTAT) [33] shows that 41.4% of sexual minority respondents say that being gay or bisexual has been a disadvantage in terms of career and professional growth, recognition and appreciation, or income and salary. In addition, 74.3% say that they have avoided expressing their sexual orientation for fear of threats or aggression, and 8.8% report that they have experienced aggression because of their sexual orientation in the last 3 years.

### The Present Study

The present research aims to assess the association between attachment anxiety and avoidance and proximal minority stressors (perceived stigma and internalized homonegativity) in a sample of Italian lesbian and gay people. According to the data discussed above, the following hypotheses are proposed:

- Attachment anxiety is positively associated with perceived stigma and internalized homonegativity;
- Attachment avoidance is positively associated with internalized homonegativity.

Given the ambiguity of the results regarding the association between attachment avoidance and proximal minority stressors, no clear hypotheses can be made in this regard.

## 2. Materials and Methods

### 2.1. Participants and Procedure

The study’s procedures conform with the ethical standards of the APA [34] and the 1964 Declaration of Helsinki [35]. The snowball sampling technique was used to gather the sample. Several associations working to protect the rights of sexual minority people were contacted to inform and invite their members to participate. Employing the LimeSurvey platform, the questionnaire was administered online. Data were collected from the end of September 2021 to April 2022 in Italy. In accordance with Italian privacy law no. 675/96, each participant signed a written informed consent, giving their consent to the use of the data. Participants were informed that they could end their participation in the study at any time, and all questionnaires were anonymous. A dedicated email address was created and made available to participants in case of questions or doubts. Each questionnaire was scored following the guidelines provided by the instrument’s author(s). The University of Turin’s Bioethical Committee has approved the research project (protocol number 0429348). A part of this dataset was analyzed and presented in a conference paper [36] in limited form. In the current study, the analyses include a larger sample and test different models using different control variables.

To be enrolled in the present research, participants had to be (A) over 18 years old, (B) native Italian speakers, and (C) self-identify as lesbian or gay. A total of 163 participants (60.7% men) aged between 20 and 76 years (*M_age_* = 32.56, *SD* = 10.87) met the inclusion criteria and completed the self-report questionnaires. The socio-demographic characteristics of the respondents are listed in Table 1.

### 2.2. Instruments

*Adult Attachment.* The Experiences in Close Relationships Scale 12 (ECR-12) [37] was used to assess adult attachment (avoidance and anxiety). The ECR-12 includes 12 items evaluated on a 7-point Likert scale from strong agreement to strong disagreement. Six of its items evaluate attachment anxiety—(e.g., “I worry a fair amount about losing my partner”), while the remaining six evaluate attachment avoidance (e.g., “I don’t feel comfortable opening up to romantic partners.”). In the current research, the reliability for attachment anxiety and attachment avoidance measured through Cronbach’s alpha was, respectively, 0.87 and 0.91.

*Perceived stigma.* Perceived stigma was assessed using the Minority Stress Scale (MSS) [38]. The MSS includes 43 items assessing proximal minority stressors and distal minority stressors. It comprises eight subscales: structural stigma (“Because of my sexual orientation, I won’t be able to have a relationship that is legally recognized”), *enacted stigma* (“Because of my sexual orientation, I have been discriminated against”) *expectations of discrimination* (“Because of my sexual orientation, I expect to be the target of insults”), *expectations of discrimination by family members* (“Because of my sexual orientation, I expect to be discriminated against by my family”), internalized homophobia towards oneself (“I am ashamed of being attracted to persons of my own sex”), internalized homophobia towards others (“I feel intense discomfort seeing a masculine woman”), *concealment of sexual orientation* (“My father knows I am gay”), and *stigma awareness* (“Because of my sexual orientation, I might be considered a pervert”). In the present study, only the *expectation of discrimination* subscale was included as measure of perceived stigma (12 items). Participants rated each item on a 5-point Likert scale ranging from 1 (*completely disagree*/*never*) to 5 (*completely agree*/*always*). The reliability (Cronbach’s alpha) of the *expectation of discrimination* subscale was 0.91.

*Internalized homonegativity*. Internalized homonegativity was evaluated with the Measure of Internalized Sexual Stigma for Lesbian and Gay Men (MISS-LG; 6-item version) [39]. The items (e.g., “after sexual intercourse with a person of the same sex I feel a strong sense of discomfort”) are rated using a 5-point Likert scale, from *totally disagree* to *totally agree*. In the present research, the reliability measured through Cronbach’s alpha was 0.75.

The following socio-demographic variables were controlled for: age, sex, and socioeconomic condition. 

### 2.3. Data Analysis

The analysis was performed using the software IBM SPSS version 29. The characteristics of the sample involved in the present study were described using descriptive analysis and frequencies. Pearson’s correlation (*r*) was used to test the association between the variables included in the study design, and Cohen’s [40] conventions were used to interpret the results found. To test whether there were sex differences in the scores of the study variables, a series of *t*-tests were performed. The reliability of the instruments used in the present research was measured through Cronbach’s alpha.

Two multiple linear regression models were applied to assess the association between attachment anxiety and avoidance (independent variables) and internalized homonegativity and perceived discrimination (independent variables). Age, sex, and socioeconomic condition were included as control variables. Multicollinearity was assessed with the variance inflation factor (VIF) with a cut-off value of 2.5. The R^2^ statistic was used to assess the fit of the models.

## 3. Results

Bivariate correlations between the variables included in the study are presented in Table 2. Attachment anxiety and avoidance were positively correlated, and both correlated positively with internalized homonegativity and perceived stigma. Perceived stigma and internalized homonegativity were positively correlated. Results on *t*-tests revealed no significant sex differences on the scores regarding adult attachment (anxiety and avoidance), perceived stigma, and internalized homonegativity.

Two multiple linear regression models were applied to evaluate the association between the variables included in the study. In the first multiple linear regression model (see Table 3), age, sex, economic satisfaction, and adult attachment (anxiety and avoidance) were the independent variables; internalized homonegativity was the dependent variable. The regression model was significant (F(5, 157) = 7.348; *p* < 0.001) and explained 19% of the variance of internalized homonegativity (R^2^ = 0.190). Specifically, economic satisfaction (b = −0.192; *p* < 0.010) was negatively associated with internalized homonegativity, whereas attachment avoidance (b = 0.157; *p* < 0.050) and attachment anxiety (b = 0.283; *p* < 0.001) were positively related to internalized homonegativity. No other variables were significantly associated with internalized homonegativity. All VIF values were below 2.5.

In the second multiple regression model (see Table 4), age, sex, economic satisfaction, attachment anxiety, and attachment avoidance were entered as independent variables; perceived stigma was the dependent variable. The regression model was significant (F(5, 157) = 8.829; *p* < 0.001) and explained 21.9% of the variance in perceived stigma (R^2^ = 0.219). Specifically, age was negatively associated with perceived stigma (b = −0.326; *p* < 0.001), whereas attachment avoidance was positively associated with perceived stigma (b = 0.233; *p* < 0.010). No other variables were significantly associated with perceived stigma. All VIF values were below 2.5.

## 4. Discussion

The aim of the present study was to investigate the association between adult attachment (anxiety and avoidance) and perceived proximal minority stressors in a group of Italian gay and lesbian people. The results partially confirmed the formulated hypotheses. Specifically, both dimensions of adult attachment (anxiety and avoidance) were positively associated with internalized homonegativity. These findings are consistent with other studies on the same subject [25,26,27,28,30]. On the one hand, the association between attachment anxiety and internalized homonegativity may be explained by the tendency of individuals with higher levels of attachment anxiety to hyperactivate the attachment system. This can promote the intense expression of attachment needs and increase the accessibility of and vulnerability to negative affect and cognitions [21,23], which in turn seems to contribute to the internalization and expression of homonegative attitudes related to one’s sexual identity. Moreover, the negative self-representations associated with higher levels of attachment anxiety may promote the internalization of homonegative attitudes. These, in turn, are harmful and frustrating but consistent with these representations and thus with the expectations of LG individuals with higher levels of attachment anxiety. On the other hand, the higher levels of internalized homonegativity associated with avoidant attachment are less intuitive. Consistent with attachment theory, the tendency to deactivate the attachment system, which is typical of individuals with avoidant attachment, should defend against the emergence of attachment needs and negative perceptions and affect. However, it is possible that in particularly stressful situations, available resources are insufficient to limit access to negative cognitions and emotions [41], thereby promoting the expression of homonegative attitudes toward the self. These data are consistent with the results found in several studies on this topic [26,27,28,30], although the study by Calvo et al. [25] found that the relationship between attachment avoidance and internalized homonegativity was fully mediated by social support. Further studies are needed, also using more complex statistical models, as some of these relationships have only been tested at the bivariate level [26,27,30].

Furthermore, in the present research, higher levels of attachment avoidance, but not attachment anxiety, predicted higher levels of perceived stigma. The positive association between attachment avoidance and perceived stigma seems to be at odds with the study by Zakalyk and Wei [29], in which higher levels of attachment avoidance predicted lower levels of perceived stigma. However, this relationship was no longer significant when the anxious dimension of adult attachment was removed from the model. As stated by the authors [29], this could be due to a partial overlap between the two dimensions of adult attachment and the intervention of moderating factors, which they identify primarily in positive self-representations and resources available to these individuals that are useful for deactivating the attachment system. This would protect against the emergence and expression of negative affect and cognitions such as perceived stigma, explaining the negative association emerged. Although these hypotheses are intriguing and deserve further investigation, it is important to note that several studies have shown that individuals with higher levels of attachment avoidance have negative perceptions of others [22,42,43]. This could promote the perception of a discriminatory and stigmatizing environment, especially in stressful situations where the deactivation strategies of the attachment system are not sufficient to limit access to these negative perceptions of others. In addition, such individuals may have difficulty in becoming intimate with others for fear of rejection. This may limit access to social support resources, which could further increase the perception of discrimination and social exclusion based on sexual orientation, paradoxically confirming expectations of rejection. Given the contradictory findings to date, future studies should further explore the relationship between attachment avoidance and perceived stigma, also assessing the role of moderator variables.

The lack of a significant relationship between attachment anxiety and perceived stigma also seems contrary to the results of the study by Zakalyk and Wei [29], which found a positive association between the two variables. The results of the present research appear to be in contrast not only with the available data, but also with the assumptions of attachment theory, which highlights a tendency for individuals with higher levels of attachment anxiety to hyperactivate the attachment system and thus be more prone to negative affect and cognitions in relation to both themselves and others [21,23]. Although these findings may be due to sampling bias, further studies are needed to investigate this relationship, as the limited data available do not allow for firm conclusions in this regard.

## 5. Limitations and Future Directions

The present study has some limitations. Firstly, the study design was cross-sectional. This does not allow any firm conclusions to be drawn about the causal direction of the associations hypothesized. It is therefore necessary to replicate the present study in studies with a longitudinal design to confirm the results obtained here.

Secondly, the sample consisted of a small number of gay and lesbian people, which makes it not representative. This limits the generalizability of the results obtained, and further studies with larger samples including other sexual identities are needed. Furthermore, given the small sample size, the analyses conducted did not allow for differentiation between gay and lesbian people, and further studies in this direction are needed to highlight differences and similarities between the two populations.

Finally, among the dimensions of minority stress, only perceived stigma and internalized homonegativity were considered. Future studies should also include sexual orientation concealment and more generally address the psychological (e.g., attachment styles and functioning, and personality traits), relational (e.g., experiences in childhood and adolescent, and family dynamics and functioning), and social factors (e.g., heterosexist attitudes and sexual discrimination) that influence individual differences in terms of perceived minority stress in order to inform interventions addressing this population.

## 6. Conclusions

The results found underscore the influence of adult attachment on levels of proximal minority stressors and provide data that support the Integrated Attachment and Sexual Minority Stress Model by Cooks and Caleb’s [24]. Although further studies in this direction are needed, especially regarding the role of attachment avoidance and the influence of adult attachment on perceived stigma, these findings provide theoretical and clinical evidence. The results seem to highlight the usefulness of interventions informed by attachment theory aimed at promoting security-based strategies of affect regulation [21] and positive representations of self and others. This could help to reduce levels of minority stress in gay and lesbian people and promote well-being in this population.

## Figures and Tables

**Table 1:** Socio-demographic characteristics of the study sample.

	*n*	*%*
Sex		
Female	60	36.8
Male	103	63.2
Gender		
Woman	59	36.2
Man	99	60.7
Transgender woman	1	0.6
Non-binary	4	2.5
Sexual Orientation		
Lesbian	60	36.8
Gay	103	63.2
Educational level ^a^		
Middle school diploma	5	3.1
High school diploma	44	27
Bachelor’s degree	42	25.8
Master’s degree or higher	39	23.9
Employment status		
Unemployed	4	2.5
Freelancer	27	16.6
Employee	69	42.3
Student	59	36.2
Homemaker	1	0.6
Retired	3	1.8
Economic satisfaction		
Unstable	18	11
Sufficient	94	57.7
Wealthy or higher	51	31.3

Note: *n* = 163. ^a^ 33 missing values.

**Table 2:** Mean scores, standard deviations, and bivariate correlations between the study variables.

	*M*	*SD*	α	1	2	3	4
1. Perceived stigma	33.61	10.12	0.91	—			
2. Internalized homonegativity	9.67	3.92	0.75	0.453 **	—		
3. Attachment anxiety	24.35	8.67	0.87	0.263 **	0.351 **	—	
4. Attachment avoidance	12.55	6.42	0.91	0.243 **	0.254 **	0.261 **	—

Note: *N* = 163. ** *p* < 0.01.

**Table 3:** Results of the multiple linear regression model predicting internalized homonegativity.

Variables	*B*	*SE*	*b*	*p*
Age	−0.008	0.028	−0.022	0.777
Sex	0.534	0.609	0.066	0.382
Economic satisfaction	−1.189	0.453	−0.192	0.009 **
Attachment anxiety	0.128	0.035	0.283	<0.001 ***
Attachment avoidance	0.096	0.047	0.157	0.042

Note: ** *p* < 0.01; *** *p* < 0.001.

**Table 4:** Results of the multiple linear regression model predicting perceived stigma.

Variables	*B*	*SE*	*b*	*p*
Age	−0.304	0.071	−0.326	<0.001 ***
Sex	−0.327	1.540	−0.016	0.832
Economic satisfaction	−1.938	1.145	−0.121	0.093
Attachment anxiety	0.146	0.089	0.125	0.103
Attachment avoidance	0.367	0.118	0.233	0.002 **

Note: ** *p* < 0.01; *** *p* < 0.001.

## Data Availability

The dataset is available upon request to the authors.
